# Falls in the general elderly population: a 3- and 6- year prospective study of risk factors using data from the longitudinal population study ‘Good ageing in Skane’

**DOI:** 10.1186/1471-2318-13-81

**Published:** 2013-08-07

**Authors:** Magnus Stenhagen, Henrik Ekström, Eva Nordell, Sölve Elmståhl

**Affiliations:** 1 Department of Health Sciences, Division of Geriatric Medicine, Lund University, Skåne University Hospital, Malmö SE-205 02, Sweden

**Keywords:** Accidental falls, Elderly, Epidemiology, Prospective, General population, Risk factors, Predictors

## Abstract

**Background:**

Accidental falls in the elderly are a major health problem, despite extensive research on risk factors and prevention. Only a limited number of multifactorial, long-term prospective studies have been performed on risk factors for falls in the general elderly population. The aim of this study was to identify risk factors predicting falls in a general elderly population after three and six years, using a prospective design.

**Methods:**

The prevalence of 38 risk factors was recorded at a baseline assessment of 1763 subjects (aged 60–93 years). The incidence of one or more falls was recorded after three and six years. The predicted risk of falling, after exposure to the various risk factors, was analysed in a multiple logistic regression model, adjusted for age and sex, and presented as odds ratios (OR). A principal component analysis (PCA), including the statistical significant factors, was also performed to identify thematic, uncorrelated components associated with falls.

**Results:**

The use of neuroleptics (OR 3.30, 95% CI: 1.15–9.43), heart failure with symptoms (OR 1.88, 95% CI: 1.17–3.04) and low walking speed (OR 1.77, 95% CI: 1.28–2.46) were prominent individual risk factors for falls. In the PCA, three main components predicting falls were identified: reduced mobility, OR 2.12 (95% CI 1.54–2.91), heart dysfunction, OR 1.66 (95% CI 1.26–2.20) and functional impairment including nocturia, OR 1.38 (95% CI 1.01-1.88).

**Conclusions:**

Three main components predicting falls were identified in a general elderly population after three and six years: reduced mobility, heart dysfunction and functional impairment including nocturia. The use of neuroleptic drugs was also a prominent individual risk factor, although the prevalence was low. Heart failure with symptoms was a significant risk factor for falls and may be of clinical importance as the prevalence of this condition in the elderly is increasing worldwide. There is need for further research on the relation between heart failure and falls in the elderly, as the treatment for this condition is poorly documented in this demographic. The findings of this study may be valuable in the development of intervention programmes aimed at sustainable, long-term reduction of falls in the elderly.

## Background

Although accidental falls in the elderly have been the subject of extensive research during the past 20 years, it is still a major health problem in a rapidly ageing global population [1-6]. Unintentional injuries are the fifth leading cause of death in older adults after cardiovascular, neoplastic, cerebrovascular and pulmonary causes. Falls are responsible for two-thirds of the fatalities resulting from unintentional injuries [2,7]. About a third of community-dwelling people over 65 years fall each year, and the incidence increases with age [1]. Approximately 20% of accidental falls require medical attention, and 5% results in a fracture or other serious injuries [8]. The additional psychological and social consequences can be severe, with post-fall syndromes including fear, depression and activity avoidance [2,7,9,10]. The high incidence of falls in the elderly, with substantial mortality and morbidity, underlines the importance of preventive interventions. Many preventive programmes and randomised controlled trials based on reported risk factors have been conducted and evaluated over the years [1,11,12]. Although some interventions have proven to be effective in reducing falls, there is still some uncertainty about the optimal approach and the efficacy of interventions aimed at preventing falls, probably due to the complex nature and aetiology of the clinical problem [2,7,8,12-15].

Risk factors for falls have been identified in epidemiological studies of varying quality [1]. Meta-studies show a notable heterogeneity in the selection, scope and methodology employed in previous studies, in which falls were assessed retrospectively, or small, unrepresentative samples of the general elderly population were studied with short follow-up periods [3,5,16,17]. As retrospective and cross-sectional studies can identify risk factors, they are methodologically weaker than prospective studies, which have the ability to assess outcome after exposure to risks [18]. Three systematic reviews of studies on risk factors for falls in subjects over 64 years of age include 23 prospective studies, from 1988 to 2009 [3,16,17]. The follow-up period ranged from 16 days to a maximum of 16 months, and most had an one-year duration. An additional, recent systematic meta-analysis included 74 prospective studies from 1988 to 2009, in which risk factors for falls in community-dwelling older people were analysed [19]. The majority of these studies had a duration of one year or less, and sample sizes less than 500 subjects. Five studies had a duration of over 36 months (range 48–84 months), although they focused on one or few risk factors, and had skewed gender distributions.

As the cause of falls in the elderly is largely multifactorial, it is relevant to analyse a broad range of intrinsic and extrinsic risk factors for falls [7,20]. Saari (2007) and Pluijm (2006) applied a multifactorial approach with 10- and 3-year follow-ups, respectively [21,22]. Anstey (2006) employed an 8-year prospective design in studying the relation between cognitive performance and falls [23]. Besides these, to our knowledge, few long-term, multifactorial prospective cohort studies on risk factors for falls in the general elderly population have been carried out. Such long-term prospective studies are vital if we are to understand the processes behind falling, to be able to predict and define groups at risk, and to improve the design of future clinical trials [18]. As the first step in an effective intervention programme may be identifying risk factors for falls, their identification in a long-term perspective may be vital in future interventions aimed at a stable, long-term reduction of falls.

This study is based on a large cohort from the general elderly population, including the very elderly, with long-term follow-up assessments. The aim of this study was to identify risk factors predicting falls in a general elderly population after three and six years. The results are also presented in terms of thematic components, using a multifactorial approach in which a wide range of risk factors for falls was analysed.

## Methods

### Study population

**Figure 1:**
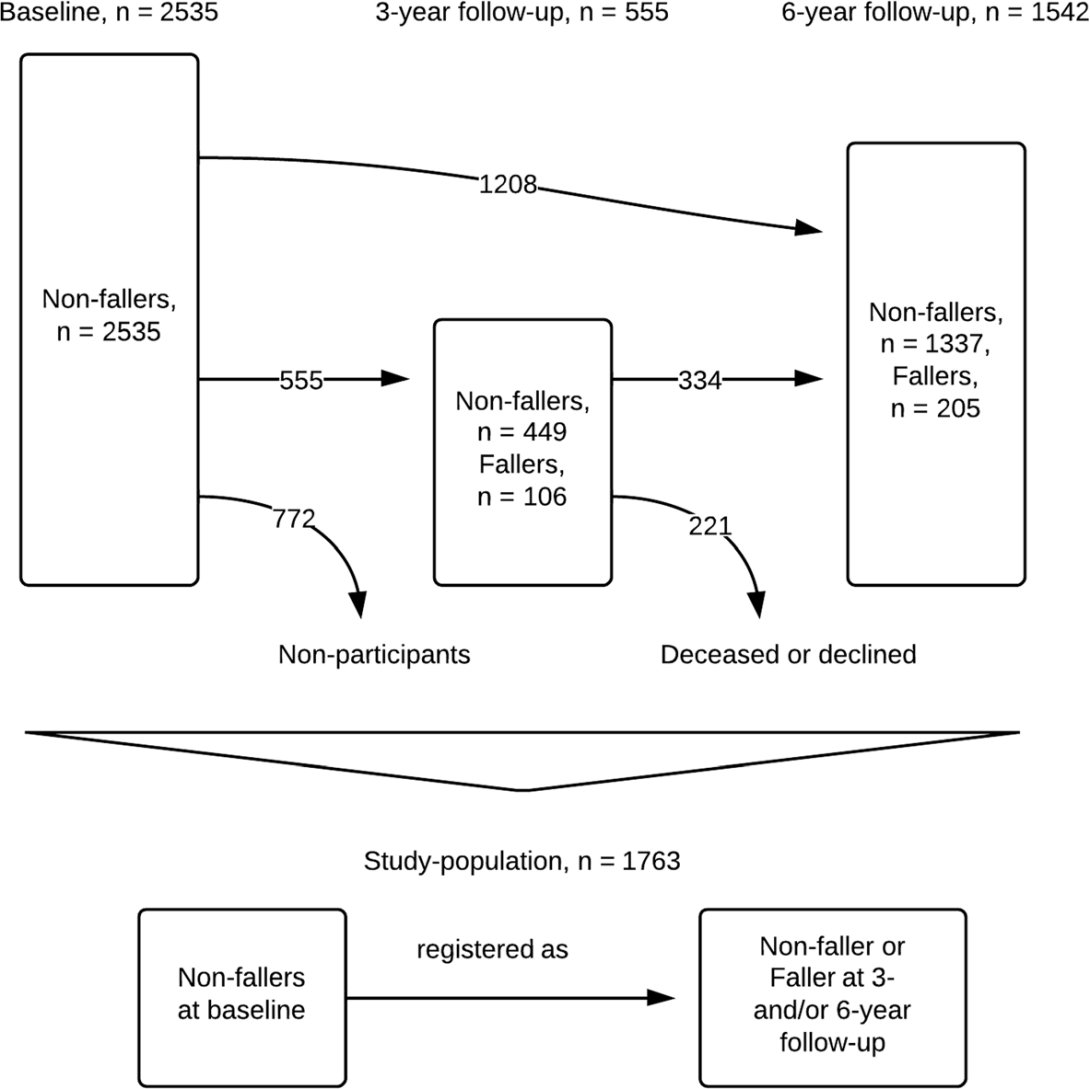
Flow sheet explaining the enrolment of subjects and the numbers participating at the 3- and 6-year follow-up assessments.

This a prospective cohort study based on data from the on-going longitudinal, Swedish population study ‘Good Ageing in Skåne’ [24]. Initially, 5370 subjects from five municipalities, covering both urban and rural areas, in the region of Skåne, southern Sweden, were invited to participate by letter. The subjects were randomly selected from the National Population Register using a computerised random number generator. The only exclusion criterion was the inability to speak Swedish. The study included men and women from nine age cohorts: 60, 66, 72, 78, 81, 84, 87, 90 and 93 years. Nearly 3000 individuals (2931) agreed to participate, giving a response rate of 60%. These subjects were recruited to a baseline assessment which took place from February 2001 to July 2004; a lengthy inclusion period was needed due to the scope of the study. The assessments were performed at a research centre or at the subject’s own home (9.7%), or in sheltered housing (2.5%). The older cohorts, 78 years and older, were invited to a 3-year follow-up assessment between January 2005 and June 2006. All cohorts were invited to a 6-year follow-up between March 2007 and December 2011. Figure 1 illustrates the study population, which initially consisted of 2535 subjects in which no falls were recorded at the baseline assessment. After recruitment, 772 subjects did not participate in the follow-up assessments, and were thus categorised as non-participants. In total, 1763 subjects with complete data from the 3- or/and 6-year follow-up assessments were included in our study.\

### Data collection and definition of variables

At baseline and the follow-up assessments, the subjects underwent a comprehensive health examination by a physician and other trained medical staff. A physical examination and cognitive assessment were carried out, their medical history was obtained, and diagnoses and medication were recorded. Self-reported questionnaires were used to obtain data on functional, physical, psychological and life-style factors.

Systematic reviews and meta-analyses have identified a number of well-established risk factors for falls: advanced age, female gender, gait and balance deficits, muscle weakness, low walking speed, dizziness and vertigo, mobility limitations and the use of assistive devices, living alone, pain, functional limitations with dependence in activities of daily living (ADL), assisted toileting, cognitive impairment and confusion [2,7,11,16,19]. Medical conditions such as cardiovascular disease and heart failure, diabetes, arthritis, dementia, depression and incontinence are also established risk factors [11,16,19]. Additionally, the use of medication, a highly adjustable risk factor, is associated with falls in the elderly, especially psychoactive, neuroleptic and anticholinergic drugs [11,16,19,25-28]. The included factors have been divided into established categories: medical and psychological factors, medication use, sensory and neuromuscular factors, balance and mobility factors, and environmental and sociodemographic factors [29]. In an attempt to cover these areas, 38 intrinsic and extrinsic factors were included and recorded as exposure variables at the baseline assessment. These variables have been defined in a previous cross-sectional study of the baseline population [30].

#### Falls

Purpose-trained physicians collected data on falls, the dependent variable, at face-to-face interviews with the subjects at the 3- and 6-year assessments. Using a structured questionnaire, the subject was asked: “Have you fallen once or more in the last six months?” No standardised definition of a fall was used [31,32]. Those who reported one or more falls in the preceding six months were dichotomised as ‘fallers’. The occurrence, frequency or severity (e.g. requiring medical attention) of the event was not included in this dichotomised approach.

#### Medical and psychological factors

The categorisation of somatic diseases was based on the International Classification of Diseases (ICD-10) criteria after examination by a physician. The factor heart disease included angina, myocardial infarction and arrhythmia. Heart failure with symptoms was identified using the New York Heart Association (NYHA) criteria, and included subjects with NYHA class II-IV symptoms [33]. The factor stroke included cerebral infarction, haemorrhage and transient ischaemic attack. Anaemia was classified using a venous blood sample (Hb <134 g/L for men, Hb <117 g/L for women) [34]. The criteria in the Diagnostic and Statistical Manual of Mental Disorders (DSM-IV) were used for the psychiatric diagnoses depression and psychosis, in an examination by a physician. A score below 24 points on the Mini-Mental State Examination scale defined cognitive impairment [35]. The prevalence of sleeping disorders was assessed using a self-reported questionnaire.

#### Medication use

The subject’s on-going medication was recorded at baseline by a physician, and categorised according to the Anatomical Therapeutic Chemical classification system [36]. The use of sedatives/hypnotics, neuroleptics, anticholinergic drugs, antihypertensive drugs (beta-blockers, ACE inhibitors, calcium-channel blockers) and diuretics (thiazide and loop diuretics) was included as risk factors, based on the results of previous meta-studies [27,37-39]. Additionally, the use of one or more of these medications was categorised as the factor ‘use of fall risk drugs’, while the use of other drugs apart from these was categorised as ‘use of other drugs’.

#### Sensory and neuromuscular factors

Vertigo, current pain, urine incontinence and nocturia were assessed using self-reported questionnaires.

#### Balance and mobility factors

Information on a self-perceived tendency to fall and pain during movement was obtained from a self-reported questionnaire. Impaired mobility was defined as using a walking aid. Walking speed was measured by timing the subject’s maximal walking speed over 15 metres without running. Low walking speed was defined as a time above the median value of the study population. A score of one or two on the six-level Mattiasson–Nilo scale was classified as low physical activity [40].

#### Environmental and sociodemographic factors

Information about the subject’s housing situation was obtained from a questionnaire. Residential home was defined as living in sheltered housing, or in a retirement or nursing home. Outdoor adaptation of housing was defined as the provision of improved accessibility for people with mild or severe disabilities, or those in wheelchairs. Indoor adaptation was defined as having thresholds removed, the bath replaced by a shower, all rooms being made accessible by wheelchair, and/or the installation of a contact alarm or a stair-lift. Age and gender were recorded at recruitment to the study.

Functional ability was assessed by self-reporting on Hulter-Åsberg’s ADL scale [41,42]. Nine activities were divided into instrumental activity of daily living (iADL, e.g. transportation, shopping) and personal activity of daily living (pADL, e.g. eating, dressing). Dependence in iADL was defined as independency of activities of pADL with dependency in at least one activity of iADL. Dependence in pADL was defined as dependency in a least one activity of pADL. Hulter-Åsberg’s ADL scale has been evaluated for both reliability and validity [41,42].

### Statistical analysis

All variables were coded into dichotomised values except age, which was categorised according to the included cohorts: 60, 66, 72, 78, 81, 84, 87, 90 and 93 years. The association between exposure to the individual risk factor at baseline and the incidence of falls after three or six years was statistically analysed using crude odds ratios (OR). Multiple logistic regression analysis, adjusted for age and sex, was used to calculate the predicted risk of falling, and was presented as the OR to fall when exposed to the particular risk factor.

As a large set of variables was analysed in this study, principal component analysis (PCA) was used to group significant factors into smaller thematic and manageable components. This approach was used to reduce collinearity and to produce a smaller number of linear, uncorrelated components associated with falls, with all of the variance in the variables being used. The significant factors identified by multiple regression analysis adjusted for age and sex, were subjected to this analysis. Prior to performing PCA, the suitability of the data was assessed, as described in the Results section. The factors in each retained component were dichotomised into a ‘dummy-variable’, representing the whole component. The ORs for falling for these components were analysed in a final multiple logistic regression model, with the components adjusted for each other.

An attrition analysis was carried out to examine and compare the non-participants (n = 772) with the participants. This analysis was based on data collected on the subjects recruited at baseline who did not participated in the follow-up assessments for various reasons. The chi-squared test was used to analyse statistical differences between the groups.

The SPSS software package version 18 was used for all statistical analyses. A 95% confidence interval (CI), and a p-value <0.05 defined statistical significance.

### Ethics

The study was approved by the Ethics Committee of Lund University, Sweden, and all subjects gave their written informed consent.

## Results

The study population included 45.7% men and 54.3% women. Just over half the study population was in their sixties at baseline (55.5%). Approximately one in five was in their seventies or eighties (19.9% / 21.5%), and only 3.1% was in their nineties. Table 1 presents data on the incidence of falls and the basic characteristics of those defined as fallers. At the 3-year follow-up assessment, including the older subjects (78 years and older at baseline), the incidence of falls in the past six months was almost one in five (19.1%). At the 6-year follow-up assessment, almost one in seven of the participants had fallen once or more in the past six months (13.3%). Falls were almost four times more common in 90-year-olds than in 60-years olds at the 6-year assessment, and women fell almost twice as often as men throughout the study. The prevalence of risk factors at baseline, and their association with falls are presented in Table 2. Eleven factors were statistically significant in the age- and sex-adjusted regression model (indicated by boldface in the far right-hand column of Table 2), with ORs for falling ranging from 1.35 to 3.30. The use of neuroleptics (OR 3.30, 95% CI 1.15–9.43), heart failure with symptoms (OR 1.88, 95% CI 1.17–3.04), and low walking speed (OR 1.77, 95% CI 1.28–2.46) were the most prominent risk factors.

**Table 1:** Basic characteristics of fallers at the 3- and 6-year follow-up assessments, n = 1763

	**3-year follow-up**		**6-year follow-up**	
	**n, faller/total**	**%**	**n, faller/total**	**%**
**All**	106/555	19.1	205/1542	13.3
**Age in decades**				
60	-	-	106/979	10.8
70	21/159	13.2	40/303	13.2
80	68/343	20.0	47/230	20.4
90	17/53	32.1	12/30	40.0
**Sex**				
Men	34/230	14.8	68/716	9.5
Women	72/325	22.2	137/826	16.6

**Table 2:** Prevalence of risk factors and their association with falls according to adjusted multiple regression analysis, expressed as odds ratios (OR), n= 1763

**Risk factors**		**Non-fallers**	**Fallers**	**Crude OR**	**OR adjusted for age**	**OR adjusted for age and sex**
		**n / %**	**n / %**	**(95% CI)**	**(95% CI)**	**(95% CI)**
***Medical and psychological***						
Heart disease	No	1169/85	203/15	**1.70**	1.26	**1.36**
	Yes	299/77	88/23	(1.28 - 2.24)	(0.94 - 1.69)	(1.00 - 1.83)
Heart failure with symptoms	No	1369/84.5	252/15.5	**2.81**	**1.76**	**1.88**
	Yes	60/65.9	31/34.1	(1.78 - 4.42)	(1.10 - 2.83)	(1.17 - 3.04)
Stroke	No	1368/84.3	255/15.7	**2.03**	1.38	1.51
	Yes	98/72.6	37/27.4	(1.36 - 3.03)	(0.91 - 2.10)	(0.98 - 2.30)
Chronic obstructive pulmonary disease	No	1441/83.5	285/16.5	1.45	1.32	1.33
	Yes	28/77.8	8/22.2	(0.65 - 3.20)	(0.59 - 2.97)	(0.59 - 2.99)
Diabetes, type 1	No	1455/83.3	291/16.7	0.50	0.54	0.50
	Yes	10/90.9	1/9.1	(0.06 - 3.92)	(0.07 - 4.40)	(0.06 - 4.17)
Osteoporosis-related fracture	No	1330/84.1	252/15.9	**1.53**	1.01	0.85
	Yes	135/77.6	39/22.4	(1.04 - 2.23)	(0.68 - 1.51)	(0.56 - 1.29)
Hip fracture	No	1432/83.7	279/16.3	**2.44**	1.59	1.53
	Yes	21/67.7	10/32.3	(1.14 - 5.25)	(0.72 - 3.54)	(0.68 - 3.43)
Osteoarthritis of hip and/or knee	No	1230/85.0	217/15.0	**1.80**	**1.55**	**1.54**
	Yes	240/75.9	76/24.1	(1.34 - 2.41)	(1.14 - 2.10)	(1.14 - 2.10)
Dementia	No	1396/83.9	268/16.1	1.93	1.21	1.30
	Yes	27/73.0	10/27.0	(0.92 - 4.03)	(0.56 - 2.59)	(0.60 - 2.82)
Epilepsy	No	1452/83.4	289/16.6	0.39	0.35	0.33
	Yes	13/92.9	1/7.1	(0.05 - 2.97)	(0.04 - 2.80)	(0.04 - 2.63)
Parkinson’s disease	No	1462/83.5	289/16.5	1.01	0.45	0.52
	Yes	5/83.3	1/16.7	(0.12 - 8.69)	(0.05 - 3.93)	(0.06 - 4.59)
Anaemia	No	1343/83.8	260/16.2	1.54	0.96	1.26
	Yes	77/77.0	23/23.0	(0.95 – 2.50)	(0.58 – 1.58)	(0.75 – 2.13)
Depression	No	1201/84.2	226/15.8	1.32	**1.50**	1.35
	Yes	241/80.1	60/19.9	(0.96 - 1.82)	(1.08 - 2.08)	(0.97 - 1.88)
Psychosis	No	1426/83.4	283/16.6	1.22	1.40	1.37
	Yes	33/80.5	8/19.5	(0.56 - 2.67)	(0.62 - 3.14)	(0.61 - 3.08)
Cognitive impairment	No	1330/84.4	246/15.6	**2.03**	1.32	1.32
	Yes	101/72.7	38/27.3	(1.37 – 3.03)	(0.87 – 2.01)	(0.87 – 2.02)
Sleeping disorders	No	939/85.4	161/14.6	**1.46**	**1.40**	1.30
	Yes	528/80.0	132/20.0	(1.13 - 1.88)	(1.08 - 1.82)	(0.99 - 1.69)
***Medication use***						
Sedative and hypnotic drugs	No	853/82.5	181/17.5	**1.70**	1.35	1.28
	Yes	183/73.5	66/26.5	(1.23 - 2.35)	(0.97 - 1.89)	(0.91 - 1.79)
Neuroleptic drugs	No	1026/81.0	241/19.0	2.55	**3.62**	**3.30**
	Yes	10/62.5	6/37.5	(0.92 - 7.10)	(1.27 - 10.36)	(1.15 - 9.43)
Anticholinergic drugs	No	998/80.6	240/19.4	0.77	0.65	0.66
	Yes	38/84.4	7/15.6	(0.34 - 1.74)	(0.28 - 1.49)	(0.29 - 1.53)
Antihypertensive drugs	No	918/81.0	215/19.0	1.16	1.08	1.16
	Yes	118/78.7	32/21.3	(0.76 - 1.76)	(0.70 - 1.65)	(0.75 - 1.79)
Diuretic drugs	No	736/82.8	153/17.2	**1.51**	1.16	1.19
	Yes	300/76.1	94/23.9	(1.13 - 2.01)	(0.86 - 1.57)	(0.87 - 1.61)
Use of fall risk drugs	No	983/87.1	145/12.9	**2.06**	**1.50**	**1.48**
	Yes	487/76.7	148/23.3	(1.60 - 2.66)	(1.15 - 1.97)	(1.13 - 1.94)
Use of other drugs	No	921/82.6	194/17.4	0.86	0.99	0.96
	Yes	549/84.7	99/15.3	(0.66 - 1.12)	(0.75 - 1.30)	(0.73 - 1.27)
***Sensory and neuromuscular***						
Vertigo	No	1018/85.5	173/14.5	**1.56**	**1.44**	**1.36**
	Yes	452/79.0	120/21.0	(1.21 – 2.02)	(1.10 – 1.87)	(1.04 – 1.78)
Current pain	No	663/85.3	114/14.7	**1.32**	**1.34**	1.29
	Yes	787/81.6	178/18.4	(1.02 – 1.70)	(1.03 – 1.75)	(0.99 – 1.68)
Urine incontinence	No	1234/84.9	219/15.1	**1.89**	**1.49**	1.31
	Yes	200/74.9	67/25.1	(1.38 - 2.58)	(1.08 - 2.06)	(0.94 - 1.82)
Nocturia	No	1366/84.5	251/15.5	**2.80**	**1.95**	**1.75**
	Yes	68/66.0	35/34.0	(1.82 - 4.30)	(1.25 - 3.05)	(1.12 - 2.75)
***Balance and mobility***						
Tendency to fall	No	1335/84.1	253/15.9	**1.58**	1.28	1.27
	Yes	134/77.0	40/23.0	(1.08 – 2.30)	(0.87 – 1.90)	(0.86 – 1.88)
Pain during movement	No	621/85.5	105/14.5	**1.31**	**1.39**	1.30
	Yes	849/81.9	188/18.1	(1.01 – 1.70)	(1.06 – 1.82)	(0.99 – 1.70)
Impaired mobility	No	1343/85.2	233/14.8	**3.03**	**1.65**	**1.50**
	Yes	101/65.6	53/34.4	(2.11 - 4.34)	(1.11 - 2.45)	(1.00 - 2.24)
Low walking speed	No	851/90.4	90/9.6	**2.97**	**2.02**	**1.77**
	Yes	554/76.1	174/23.9	(2.25 - 3.91)	(1.47 - 2.78)	(1.28 - 2.46)
Low physical activity	No	1227/84.9	219/15.1	**1.76**	1.28	1.32
	Yes	223/76.1	70/23.9	(1.30 - 2.39)	(0.93 - 1.76)	(0.95 - 1.83)
***Environmental and sociodemographic***						
Living alone	No	931/87.2	137/12.8	**1.97**	**1.46**	1.29
	Yes	539/77.6	156/22.4	(1.53 - 2.53)	(1.12 - 1.91)	(0.98 - 1.71)
Residential home	No	1444/83.7	281/16.3	**3.74**	1.77	1.81
	Yes	11/57.9	8/42.1	(1.49 - 9.38)	(0.69 - 4.53)	(0.69 - 4.72)
Outdoor adaptation	No	317/84.8	57/15.2	1.14	1.04	1.04
	Yes	999/83.0	205/17.0	(0.83 - 1.57)	(0.75 - 1.44)	(0.75 - 1.45)
Indoor adaptation	No	1099/85.9	181/14.1	**1.83**	**1.50**	**1.45**
	Yes	371/76.8	112/23.2	(1.41 - 2.39)	(1.14 - 1.97)	(1.10 - 1.91)
***Functional ability***						
Dependence in	No	1302/84.3	243/15.7	**1.89**	1.33	1.47
iADL	Yes	125/74.0	44/26.0	(1.30 – 2.73)	(0.90 – 1.96)	(0.99 – 2.18)
Dependence in	No	1131/85.4	193/14.6	**1.86**	**1.49**	**1.35**
pADL	Yes	296/75.9	94/24.1	(1.41 – 2.46)	(1.12 – 1.99)	(1.00 – 1.81)

(Bold values indicate statistical significance).

The eleven statistically significant factors were included in the PCA. Examination of the correlation matrix obtained from the PCA revealed the presence of nine coefficients of 0.3 and above, the value indicating a moderate positive relationship according to our definition. The analysis revealed four independent components with eigenvalues exceeding 1, explaining 51.7% of the variance. Inspection of the screen plot revealed a clear break after the third component. As the fourth component consisted of a single variable, a three-component solution was chosen (Table 3). Direct oblimin rotation was used to calculate the factor loadings of the variables and no correlation between the components over 0.3 was found. The first component, called reduced mobility, included the factors indoor adaptation, osteoarthritis of hip and/or knee, low walking speed and impaired mobility. It explained 21.3% of the variance and had an OR for falling of 2.12 (95% CI 1.54–2.91). The second component, consisting of heart disease, heart failure with symptoms and the use of fall risk drugs, was called heart dysfunction, and explained 11.2% of the variance with an OR for falling of 1.66 (95% CI 1.26–2.20). The final component, called functional impairment, included dependence in pADL and nocturia, and explained 9.8% of the variance, with an OR for falling of 1.38 (95% CI 1.01–1.88).

**Table 3:** Principal component analysis with eligible components and their predicted risk of falling, expressed as odds ratios (OR), n = 1763

	**Component and factor loading**		
**Risk factors**	**Reduced mobility**	**Heart dysfunction**	**Functional impairment**
Indoor adaptation	.66		
Osteoarthritis of hip and/or knee	.63		
Low walking speed	.58		
Impaired mobility	.41		
Heart disease		-.78	
Heart failure with symptoms		-.71	
Use of fall risk drugs		-.59	
Dependence in pADL			.87
Nocturia			.85
**OR for falling**	**2.12**	**1.66**	**1.38**
(95% CI)	(1.54 - 2.91)	(1.26 - 2.20)	(1.01 - 1.88)

The attrition analysis revealed that there were significantly relatively more 80- and 90-year-olds in the group of non-participants than in the included study population (Table 4). Risk factors such as heart disease, tendency to fall, the use of fall risk drugs and low walking speed were significantly more prevalent in the non-participants.

**Table 4:** Attrition analysis comparing non-participants to the study population, differences in distributions analysed with the chi-squared test

	**Non-participants**		**Study population**		
	**n = 772**		**n = 1763**		
	**n**	**%**	**n**	**%**	**p**
**Sex**					
Men	347	44.9	805	45.7	0.762
Women	425	55.1	958	54.3	0.762
**Age**, decade					
60	314	40.7	979	55.5	<0.001
70	147	19.0	350	19.9	0.664
80	222	28.8	379	21.5	<0.001
90	89	11.5	55	3.1	<0.001
**Risk factors**					
***Medical and psychological***					
Heart disease	204/766	26.6	387/1759	22.0	0.012
Heart failure with symptoms	97/740	13.1	91/1712	5.3	<0.001
Stroke	98/771	12.7	135/1758	7.7	<0.001
Chronic obstructive pulmonary disease	39/769	5.1	36/1762	2.0	<0.001
Diabetes. type 1	11/766	1.4	11/1757	0.6	0.044
Osteoporosis-related fracture	119/765	15.6	174/1756	9.9	<0.001
Hip fracture	27/760	3.6	31/1742	1.8	0.007
Osteoarthritis of hip and/or knee	152/772	19.7	316/1763	17.9	0.292
Dementia	52/733	7.1	37/1701	2.2	<0.001
Epilepsy	13/769	1.7	14/1755	0.8	0.045
Parkinson’s disease	11/764	1.4	6/1757	0.3	0.002
Anaemia	90/719	12.5	100/1703	5.9	<0.001
Depression	135/758	17.8	301/1728	17.4	0.813
Psychosis and other mental illness	18/768	2.3	41/1750	2.3	0.999
Cognitive impairment	127/695	18.3	139/1715	8.1	<0.001
Sleeping disorders	317/772	41.1	660/1760	37.5	0.090
***Medication use***					
Sedative and hypnotic drugs	158/630	25.1	249/1283	19.4	0.004
Neuroleptic drugs	17/630	2.7	16/1283	1.2	0.022
Anticholinergic drugs	34/630	5.4	45/1283	3.5	0.051
Antihypertensive drugs	85/630	13.5	150/1283	11.7	0.259
Diuretic drugs	286/630	45.4	394/1283	30.7	<0.001
Use of fall risk drugs	393/772	50.9	635/1763	36.0	<0.001
Use of other drugs	237/772	30.7	648/1763	36.8	0.003
***Sensory and neuromuscular***					
Vertigo	276/772	35.8	572/1763	32.4	0.104
Current pain	418/760	55.0	965/1742	55.4	0.855
Urine incontinence	149/690	21.6	267/1720	15.5	<0.001
Nocturia	74/690	10.7	103/1720	6.0	<0.001
***Balance and mobility***					
Tendency to fall	121/771	15.7	174/1762	9.9	<0.001
Pain during movement	441/764	57.7	1037/1763	58.8	0.607
Impaired mobility	153/700	21.9	154/1730	8.9	<0.001
Low walking speed	372/617	60.3	728/1669	43.6	<0.001
Low physical activity	238/717	33.2	293/1739	16.8	<0.001
***Environmental and sociodemographic***					
Living alone	347/772	44.9	695/1763	39.4	0.009
Residential home	28/727	3.9	19/1744	1.1	<0.001
Outdoor adaptation	496/648	76.5	1204/1578	76.3	0.902
Indoor adaptation	275/772	35.6	483/1763	27.4	<0.001
***Functional ability***					
Dependence in iADL	93/686	13.6	169/1714	9.9	0.009
Dependence in pADL	222/686	32.4	390/1714	22.8	<0.001

## Discussion

We found that the component reduced mobility was a prominent predictor of falling. This is in accordance with previous findings, where several studies have revealed a relationship between low walking speed and falls in the elderly [43,44]. Knee pain and lower back pain have also been significantly associated with multiple falls, predominantly in women [44,45]. Osteoarthritis and pain in the musculoskeletal system may reduce the ability of the individual to maintain an upright position in non-ideal surroundings, and treatment with opioids may cause additional adverse effects in the elderly, such as dizziness. Randomised trials and systematic reviews have shown that exercise, strength and balance training can significantly reduce both non-injurious and injurious falls in the elderly [1,8,13].

The association between heart dysfunction and future falls is partly a confirmatory finding, where the most common cardiovascular disorders associated with falls are carotid sinus hypersensitivity, vasovagal syndrome and brady- and tachyarrhythmias [46]. However, heart failure with symptoms was a prominent individual predictor, which may not have been widely reported. This may be clinically relevant, as heart failure is a cardiovascular disease with increasing incidence and prevalence, and in an ageing population it can be expected to rise significantly in the coming decades [47,48]. According to a previous review, there is a relative lack of published clinical trials in which the efficacy of heart failure treatment has been assessed exclusively in elderly individuals [47]. In a recent geriatric study, falls and urinary incontinence were reported as being common comorbidities in individuals with heart failure approaching the end of life, and there is limited knowledge on the best way to manage these individuals [49]. The elevated risk of falling in the elderly with heart failure may be explained by its pharmacotherapy (e.g. diuretics, ACE inhibitors, beta-blockers) and their association with falls, although these categories of pharmaceuticals did not individually predict falls in our study. Response to pharmacotherapy for heart failure varies in elderly individuals, who are susceptible to adverse events such as orthostatic hypotension, dehydration, electrolyte disturbance, incontinence and drug-drug interactions [47]. One common side effect of diuretics is hyponatraemia, which is associated with impairment of gait and attention. Stable mild chronic hyponatraemia is generally considered asymptomatic, although Renneboog (2006) observed a high number of falls in comparison to controls in hyponatraemic subjects considered clinically asymptomatic. Furthermore, individuals with moderate chronic hyponatraemia fell dramatically more frequently than patients with normal serum sodium levels [50].

The component functional impairment, including dependence in pADL and nocturia, predicted falls, and has previously been reported to be a risk factor [2,4,51]. In the limited studies of fall risk factors in the very elderly, dependence in pADL was found to be one of the most important independent predisposing factors in a population-based study including individuals 85–103 years old [4]. In a recent study, the elevated risk of falling in adults over 85 years appeared to be due to the overall deterioration of health with age. Among those with excellent overall health, the risk of falling in adults over 85 years was no higher than in those 65–84 years of age [52]. The association between nocturia and falls may be explained by incontinence as a symptom of a general deterioration of health. Night-time visits to the toilet in darkness may also contribute to the risk of falling [53]. There may also be an association between nocturia and the treatment for heart failure, as these groups both showed an elevated risk of falling. As diuretic drugs can cause urge incontinence, orthostatic hypotension and hyponatraemia, their relation to nocturia and night-time falls may be underestimated.

The use of neuroleptics, although the prevalence is low, is found to be a prominent individual risk factor for falls in the present study. Although they have not been as widely studied in this field as other psychotropic and psychoactive drugs, such as SSRIs and sedatives, an association between neuroleptics and falls has been reported previously [27,39,54]. Unnecessary or incorrect use of drugs is one of the most easily correctable risk factors for falls in the elderly, and it was shown in a randomised trial that gradual withdrawal of psychotropic drugs reduced the risk of falling by 66% [55].

### Strengths and limitations

This is a long-term, prospective cohort study of a relatively large sample of a general, elderly population, which can be seen as a strength. A prospective design, allowing the study of outcome following exposure, ranks higher in the hierarchy of evidence, than retrospective or cross-sectional studies [18]. Studying the effect of risk factors on falls over an extended period may provide valuable information for future fall-prevention programmes. As a long-term, persistent reduction of falls are desirable, an intervention on risk factors identified in long-term prospective studies, may results in sustainable reduction of falls in the elderly. Although this is a prospective study of risk factors for falls, the direct causality of falls cannot be identified using this epidemiological approach. The risk factors predicting falls, described in this study, are rather markers of an increased probability of a future fall.

Unfortunately, selection bias cannot be ruled out in this study, despite the fact that home visits were offered to those who were unable to come to the research centre. Older and more infirm subjects did not participate in the study to the same extent as younger and healthier individuals. The attrition analysis confirmed that there were significantly relatively more older, and assumingly frailer, subjects in the group of non-participants than in the study population. The majority of the fall risk factors considered in this study had a significantly higher relative prevalence in the non-participants than in those included in the study. A selection bias, leading to underrepresentation of older and frailer individuals, and diagnoses such as stroke and dementia, may have reduced the predicted fall risk in this study.

The reported incidence of falls during the past six months for the older cohorts at the 3-year follow-up assessment (19.1%), and at the 6-year follow-up assessment (13.3%) are low compared to those reported in previous studies [1,2,7,11]. Apart from selection bias and attrition after six years, the low incidence observed here may be due to a general better health in the population studied, than previously assumed. Furthermore, the gender distribution was very equal in the study population. Previous studies of risk factors associated with falls have shown a tendency towards the recruiting of more women than men, which may introduce bias, as women are more prone to fall than men [19].

The long-term design of this study leads to a number of methodological limitations in recording falls, which will affect the reliability and validity of the dependent variable. The Prevention of Falls Network Europe (ProFaNE) collaborators’ recommendation of weekly, monthly or bi-monthly recording of falls in prospective studies was not possible in this epidemiological study [31,32]. Falls six months prior the follow-up assessments were recorded, which means there was a relatively long period during which falls or other adverse events could have taken place. It was not possible to specify the occurrence, severity or frequency of falls with the dichotomized approach used here. Falls were recorded by purpose-trained physicians in face-to-face interviews, using a structured questionnaire. The recommended ProFaNE definition of a fall [31,32] was not used. Although there is no universally accepted method of reporting falls, and the elderly may intuitively give correct information, this is a limitation of the study. As falls are common in the elderly population, our approach may have led to underreporting and misclassification of these events. Despite this, the likelihood of this possible bias producing false positive conclusions would have been limited. As the incidence of falls is very likely to be underreported, it rather reduces the significance and strength of the results throughout the study.

## Conclusions

Three main components predicting falls were identified in a general elderly population after three and six years: reduced mobility, heart dysfunction, and functional impairment including nocturia. Furthermore, the use of neuroleptic drugs, although low in prevalence, was a prominent individual risk factor. Heart failure with symptoms was a significant risk factor for falls, and may be of clinical importance as the prevalence in the elderly is increasing worldwide. There is need for further research on the relation between heart failure and falls in the elderly, as the treatment of the condition is poorly documented in this demographic. The findings of this study may be valuable for future trials aimed at sustainable, long-term reduction of falls in the elderly.

## Competing interests

The authors declare that they have no competing interest.

## Authors’ contributions

MS drafted the manuscript and was the main author. MS, HE, EN and SE took part in the methodological decisions, statistical analyses and interpretation of data, commented on the draft, and all authors read and approved the final version. SE was the main planner of the study.

## Pre-publication history

The pre-publication history for this paper can be accessed here:

http://www.biomedcentral.com/1471-2318/13/81/prepub
